# Oxidative stress in the brain causes hypertension via sympathoexcitation

**DOI:** 10.3389/fphys.2012.00335

**Published:** 2012-08-17

**Authors:** Takuya Kishi, Yoshitaka Hirooka

**Affiliations:** ^1^Department of Advanced Therapeutics for Cardiovascular Diseases, Kyushu University Graduate School of Medical SciencesFukuoka, Japan; ^2^Department of Advanced Cardiovascular Regulation and Therapeutics, Kyushu University Graduate School of Medical SciencesFukuoka, Japan

**Keywords:** brain, oxidative stress, sympathetic nerve activity, hypertension, angiotensin II

## Abstract

Activation of the sympathetic nervous system (SNS) has an important role in the pathogenesis of hypertension, and is determined by the brain. Previous many studies have demonstrated that oxidative stress, mainly produced by angiotensin II type 1 (AT_1_) receptor and nicotinamide adenine dinucleotide phosphate (NAD (P) H) oxidase, in the autonomic brain regions was involved in the activation of the SNS of hypertension. In this concept, we have investigated the role of oxidative stress in the rostral ventrolateral medulla (RVLM), which is known as the cardiovascular center in the brainstem, in the activation of the SNS, and demonstrated that AT_1_ receptor and NAD (P) H oxidase-induced oxidative stress in the RVLM causes sympathoexcitation in hypertensive rats. The mechanisms in which brain oxidative stress causes sympathoexcitation have been investigated, such as the interactions with nitric oxide (NO), effects on the signal transduction, or inflammations. Interestingly, the environmental factors of high salt intake and high calorie diet may also increase the oxidative stress in the brain, particularly in the RVLM, thereby activating the central sympathetic outflow and increasing the risk of hypertension. Furthermore, several orally administered AT_1_ receptor blockers have been found to cause sympathoinhibition via reduction of oxidative stress through the inhibition of central AT_1_ receptor. In conclusion, we must consider that AT_1_ receptor and the related oxidative stress production in the brain cause the activation of SNS in hypertension, and that AT_1_ receptor in the brain could be novel therapeutic target of the treatments for hypertension.

## Introduction

Recent many studies have suggested that sympathetic nervous system (SNS) is important in the pathogenesis, initial pathological events, development, and end organ damages of hypertension (Grassi, 2009, 2010; Esler, 2010; Grassi et al., 2010; Mauo et al., 2010). The activation of the SNS is determined by brain, especially by a rostral ventrolateral medulla (RVLM) in the brainstem (Dampney, 1994; Guyenet, 2006). The RVLM neurons determine the basal central sympathetic outflow and integrate the inputs from baroreceptors, chemoreceptors, and visceral receptors via the nucleus of the solitary tract (NTS) (Pilowsky and Goodchild, 2002; Dampney et al., 2003; Sved et al., 2003; Campos and Bergamschi, 2006; Guyenet, 2006; Carlson and Wyss, 2008), and it also receives the inputs from the paraventricular nucleus (PVN) of the hypothalamus, which is known as a key nucleus of the central cardiovascular regulation (Pilowsky and Goodchild, 2002; Dampney et al., 2005; Coote, 2007). Therefore, RVLM is known as a cardiovascular center.

The importance of systemic oxidative stress has already been determined in the various aspects of hypertension (Briones and Touyz, 2010). There are several studies with regard to the role of brain oxidative stress in the activation of the SNS and hypertension (Tai et al., 2005; Peterson et al., 2006; Hirooka, 2008, 2011; Campos, 2009; Hirooka et al., 2010, 2011). Among them, we have investigated the role of oxidative stress in the brain, particularly in the RVLM, in the pathogenesis of hypertension (Kishi et al., 2004, 2008, 2009, 2010a,b, 2011a, 2012a,b; Koga et al., 2008; Konno et al., 2008, 2012; Nozoe et al., 2008; Kishi and Sunagawa, 2011, 2012; Nishihara et al., 2012). Other investigators have also confirmed our initial observations, and other details have been further investigated. In this review, we describe the importance of oxidative stress in the brainstem, particularly in the RVLM, on the regulation of the SNS and its contribution to the pathophysiology of hypertension.

## Oxidative stress in the RVLM causes sympathoexcitation in hypertensive RATs

We have demonstrated that oxidative stress in the RVLM is increased and contributes to the neural mechanisms of hypertension in stroke-prone spontaneously hypertensive rats (SHRSPs) (Kishi et al., 2004, 2008, 2009, 2010b, 2012a,b; Kishi and Sunagawa, 2011, 2012) and spontaneously hypertensive rats (SHRs) (Koga et al., 2008; Nishihara et al., 2012). We also confirmed that a microinjection of tempol, a membrane-permeable superoxide dismutase (SOD) mimetic, into the RVLM decreased blood pressure and heart rate in SHRSPs, but not in normotensive rats. Furthermore, we transfected adenovirus vectors encoding the MnSOD gene into the bilateral RVLM in SHRSPs. Overexpression of MnSOD in the RVLM decreased blood pressure, heart rate and urinary norepinephrine excretion in SHRSPs, but not in normotensive rats. We also found reduced SOD activity in the RVLM of SHRSPs compared with normotensive rats, which led to a decreased capability of scavenging superoxide anions. Together, our these findings indicate that oxidative stress in the RVLM increased blood pressure, which may have occurred via an activation of the SNS, and this mechanism was involved in the neural pathophysiology of hypertension in SHRSPs. Consistent with our observations, it was reported that increased superoxide anion in the RVLM contributed to hypertension in SHRs (Tai et al., 2005). We have also demonstrated that oxidative stress in the RVLM causes sympathoexcitaion in other several hypertensive models, such as, salt-induced hypertension (Koga et al., 2008), dietary-induced hypertension (Kishi et al., 2011a; Konno et al., 2012), and experimental jet lag (Kishi and Sunagawa, 2011). These results are consistent with previous studies of other investigators (Fujita et al., 2007; Nagae et al., 2009).

Interestingly, every intervention led to a similar extent of reduction of blood pressure and superoxide production in hypertensive rats. In our studies, the reduction of oxidative stress in the RVLM causes prominent sympathoinhibition. Overexpression of MnSOD (Kishi et al., 2004; Nishihara et al., 2012), or microinjection of tempol (Kishi et al., 2004; Koga et al., 2008; Konno et al., 2012) decreases the activation of the SNS with reduction of the oxidative stress in the RVLM. In addition, Oliveira-Sales et al suggested that sympathoexcitation in renovascular (two-kidney one-clip) hypertensive rats are associated with oxidative stress in the RVLM and PVN of the hypothalamus and with systemic oxidative stress (Oliveira-Sales et al., 2008, 2009). Because, the two-kidney and one-clip model is an angiotensin II-dependent hypertension model, it is not surprising that oxidative stress was increased in this model. However, it is important that the increased oxidative stress in the autonomic brain regions, such as, the RVLM and PVN of the hypothalamus, was involved in the activation of the SNS as one of the mechanisms of hypertension in this model. Moreover, a recent study found that oxidative stress in the RVLM had a major role in the enhanced central sympathetic outflow in the two-kidney one-clip hypertensive rats (Oliveira-Sales et al., 2010a). These results strongly suggest that the increase in oxidative stress in the RVLM is an important cause, not result, of the sympathoexcitation, which leads to hypertension.

## Sources of oxidative stress in the RVLM

As the sources of oxidative stress production in the brain, there are several candidates, such as, nicotinamide adenine dinucleotide phosphate (NAD (P) H) oxidase, xanthine oxidase, uncoupled nitric oxide synthase, and mitochondria (Hirooka, 2008, 2011; Hirooka et al., 2010, 2011). Among them, we demonstrated that the activation of the NAD (P) H oxidase through angiotensin II type 1 (AT_1_) receptor had a major role in the oxidative stress production in the RVLM of SHRSPs (Nozoe et al., 2008; Kishi et al., 2010a, 2012b). Regional expression of the NAD (P) H oxidase has been demonstrated in the brain including the NTS and RVLM (Infanger et al., 2006; Campese et al., 2007; Bai et al., 2009). Peripheral slow-pressor dose of angiotensin II in mice led to a gradual development of hypertension that was correlated with marked elevation in superoxide production (Zimmerman et al., 2004b). In this case, the authors emphasized the importance of the sub-fornical organ (SFO), in which the blood-brain barrier is lacking and AT_1_ receptor is rich. Angiotensinergic inputs in the SFO are delivered to the PVN of the hypothalamus, and then it sends the neural information to the RVLM neurons. Thus, the functional responses of the SFO to angiotensin II are the increases in drinking behavior and blood pressure via the activation of the SNS and vasopressin release. In addition, we showed that Rac1 activation occurs in the activation of NAD (P) H oxidase (Nozoe et al., 2007; Hirooka et al., 2010; Kishi et al., 2010b). Rac1 is a small G protein involved in integrating the intracellular transduction pathways toward NAD (P) H activation and requires lipid modifications to migrate from the cytosol to the cell membrane. The inhibition of Rac1 caused by the transfection of the adenovirus vectors encoding a dominant negative Rac1 into the RVLM or NTS decreased blood pressure, heart rate and urinary norepinephrine excretion in SHRSPs, but not in normotensive rats (Nozoe et al., 2007; Hirooka et al., 2010). The blockade of Rac1 translocation from cytosol to membrane in the RVLM of SHRSPs causes sympathoinhibition via inhibition of NAD (P) H oxidase and oxidative stress (Kishi et al., 2010b). It was demonstrated that Nox2-containing NAD (P) H oxidase followed by an influx of Ca^2+^ via the L-type calcium channels was the source of the angiotensin II-induced oxidative stress production in the NTS neurons that were anterograde labeled from the vagal afferents (Wang et al., 2004, 2006). Because, azelnidipine could reduce oxidative stress in the RVLM of SHRSPs associated with sympathoinhibition, the inhibition of the Ca^2+^ channel in the RVLM may have reduced oxidative stress (Konno et al., 2008; Shinohara et al., 2012) Thus, our findings indicate that the activation of the Rac1 in the RVLM or NTS produces oxidative stress via the NAD (P) H oxidase in SHRSPs. In fact, it was demonstrated that the activation of the Rac1/NAD (P) H oxidase was required in the pressor and dipsogenic actions of angiotensin II in the brain (Zimmerman et al., 2004a)

The brain renin-angiotensin system is activated in hypertension and chronic heart failure with enhanced central sympathetic outflow (Hu et al., 2002; Reja et al., 2006; Leenen, 2007; Huang and Leenen, 2009; Zucker et al., 2009; Dupont and Brouwers, 2010) Aldosterone increases angiotensin-converting enzyme, AT_1_ receptor and oxidative stress in the PVN of the hypothalamus of salt-sensitive hypertension and ischemic heart failure (Huang et al., 2011). It has been determined that mitochondria-derived oxidative stress mediates sympathoexcitation induced by angiotensin II in the brain (Nozoe et al., 2007; Zimmerman and Zucker, 2009). Exogenously administered angiotensin II into the RVLM elicits the pressor response via activation of the SNS (Hirooka et al., 1997; Dampney et al., 2007) The inhibition of AT_1_ receptor in the RVLM by an AT_1_ receptor blocker reduces blood pressure with sympathoinhibition in hypertensive rats (Hirooka et al., 1997; Koga et al., 2008; Kishi et al., 2010a, 2011a, 2012a,b; Konno et al., 2012). We found that the overexpression of MnSOD attenuated the angiotensin II-induced pressor response and also suppressed the angiotensin II-induced oxidative stress production in the RVLM (Nozoe et al., 2008) A recent study also demonstrated that the oxidative stress-induced impairment of the mitochondrial electron transport chain complexes in the RVLM contribute to further chronic oxidative stress, thereby leading to augmented central sympathetic outflow and hypertension (Chan et al., 2009). All these results indicate that AT_1_ receptor/NAD (P) H oxidase would be a main source to produce the oxidative stress in the RVLM of hypertensive rats.

## Mechanisms of brain oxidative stress-induced sympathoexcitation

There is an interaction between superoxide and nitric oxide (NO). In the brain, NO inhibits the activation of the SNS (Kishi et al., 2001, 2002; Patel et al., 2001; Hirooka et al., 2003). We found that an increase in NO in the RVLM reduces blood pressure, heart rate, and the activation of the SNS in normotensive rats and SHRSPs (Kishi et al., 2001, 2002), and the magnitude of the decreases in these variables were greater in SHRSPs than in normotensive rats, suggesting a deficiency in the NO bioavailability in SHRSPs. There is a possibility that the deficiency in the NO bioavailability might be induced by oxidative stress. Furthermore, we demonstrated that that overexpression of inducible NO synthase (iNOS) in the RVLM elicited blood pressure elevation and sympathoexcitation in normotensive rats via increase in oxidative stress (Kimura et al., 2005). This may have been caused by the so-called uncoupling of the NO synthase function because of the deficiency of the precursor of L-arginine and/or the cofactor tetrahydrobiopterin. Importantly, we have found that the iNOS expression in the RVLM is enhanced in SHRs compared with normotensive rats, and the microinjection of iNOS blockers into the RVLM reduced blood pressure only in SHRs (Kimura et al., 2009). A recent study also suggested that the up-regulation of AT_1_ receptor and iNOS in the RVLM was important for the maintenance of high blood pressure and renal sympathetic activation in the two-kidney one-clip hypertensive rats (Oliveira-Sales et al., 2010b).

In the relationship between superoxide and NO, we should focus on the peroxynitrite formation, because the kinetics formation of peroxynitrite from superoxide and NO is strong (Zielonka et al., 2012). Actually, peroxynitrite in the RVLM has an excitotoxic effect (Zanzinger, 2002). A recent study suggested that reactive oxygen species and reactive nitrogen species, such as peroxynitrite, could dose-dependently regulate iNOS function, and that peroxynitrite reduces both NO and superoxide production via enzymatically iNOS dysfunction (Sun et al., 2010). Another report indicated that an interactive action between NO and superoxide in the RVLM via formation of peroxynitrite contributes to the un-sustained hypotensive effect of NO after overexpression of endothelial NO synthase in SHRs (Kung et al., 2008). The role of peroxynitrite from NO and superoxide in the brain on the regulation of the SNS should be further examined.

In the brain, the balance between excitatory and inhibitory amino acids determines the neural activity (Li et al., 2002; Garthwaite, 2008). In hypertensive rats, inhibitory amino acid γ-amino butylic acid (GABA) in the RVLM is decreased (Kishi et al., 2002), which in part contributes to the activation of the SNS. NO in the RVLM increases GABA release (Kishi et al., 2001). We consider that oxidative stress would reduce the release of GABA in the RVLM via deficiency in NO bioavailability, which might cause sympathoexcitation. Interestingly, we recently demonstrated that oxidative stress modulates the balance between excitatory amino acid glutamate and GABA in the RVLM of hypertensive rats (Nishihara et al., 2012).

We also have focused the signal transduction associated with oxidative stress. AT_1_ receptor activates caspase-3 through the Ras/mitogen-activated protein kinase/extracellular signal-regulated kinase (ERK) in the RVLM, which is involved in the sympathoexcitation in SHRSPs (Kishi et al., 2010a,b,c). The activities of Ras, p38 mitogen-activated protein kinase (MAPK), ERK and caspase-3 in the RVLM were elevated in SHRSPs compared with those in normotensive rats. The phosphorylation of the pro-apoptotic protein Bax and Bad, which releases cytochrome *c* in the mitochondria, leads to caspase-3 activation (Kishi et al., 2010a,b,c). In contrast, the phosphorylation of the anti-apoptotic protein Bcl-2 inhibits the caspase-3 activation. Intracerebroventricular (ICV) infusion of a caspase-3 inhibitor reduces blood pressure, heart rate and the activation of the SNS in SHRSPs, but not in normotensive rats. ICV infusion of an AT_1_ receptor blocker also reduced blood pressure, heart rate, and activation of the SNS and also reduced the activities of Ras, p38 MAPK, ERK, and caspase-3 in the RVLM of SHRSPs, suggesting that these pathways exist downstream to the AT_1_ receptor activation in the RVLM of SHRSPs and are related to blood pressure elevation and sympathoexcitation of SHRSPs. In support of our findings, it was reported that the NAD (P) H oxidase derived superoxide anion mediates the activation of p38 MAPK or ERK but not the stress-activated protein kinase/Jun N-terminal kinase by angiotensin II in the RVLM and pressor response (Chan et al., 2005). Furthermore, in a later study, the authors suggested that the activation of the NAD (P) H oxidase/ERK in the RVLM induced the phosphorylation of the transcriptional factor cyclic adenosine monophosphate response element-binding protein and c-fos induction, thereby contributing to the long-term pressor response triggered by angiotensin II (Chan et al., 2007). It is also important to note that the activation of the activator protein 1 and the Jun N-terminal kinase may occur in rabbits with rapid pacing-induced heart failure (Liu et al., 2006). Thus, the signaling pathway followed by the oxidative stress production may differ between hypertension and heart failure because of the advancement of the disease state.

Recently, the further central mechanisms of sympathoexcitation associated with oxidative stress are focused, such as, perivascular macrophages in the brain (Yu et al., 2010; Hirooka, 2010), neuron-astrocyte uncoupling (Kishi et al., 2010c, 2011b), transcription factor NF-κB (Cardinale et al., 2011), or microglial cytokines (Shi et al., 2010) in the brain causes sympathoexcitation in hypertension and heart failure. Further studies are required to clarify these mechanisms.

## Influence of salt and obesity on oxidative stress in the brain and sympathetic nerve activity

A high salt intake is an important environmental factor for the development of hypertension (Adrogue and Madias, 2007). Increasing evidence suggests that central nervous system mechanisms are involved in salt-induced hypertension, although, the kidney also has a key role in salt-induced hypertension (Brooks et al., 2005; Huang et al., 2006; Adams et al., 2007; Osborn et al., 2007). We also previously demonstrated that the increased AT_1_ receptor and NAD (P) H oxidase expression levels in the RVLM were responsible for the increased oxidative stress production and blood pressure in SHRs with a high salt intake compared with those with a regular salt intake (Koga et al., 2008). Consistent with our findings, increased oxidative stress was involved in the blood pressure elevation through an enhanced central sympathetic outflow in Dahl salt-sensitive rats (Fujita et al., 2007). It is possible that the enhanced neuronal activity in the PVN of the hypothalamus would augment the RVLM neuronal activity in these concepts.

It has also been demonstrated that the activation of the SNS has an important role in obesity-related hypertension, including the metabolic syndrome (Landsberg, 2001; Rahmouni et al., 2005; Esler et al., 2006; Grassi, 2006, 2007; Stocker et al., 2007). Insulin or leptin increases the activation of the SNS in obesity or metabolic syndrome (Rahmouni et al., 2005; Prior et al., 2010; Hilzendeger et al., 2012). In addition, it has been demonstrated that the RVLM neurons are activated in obesity-induced rats (Stocker et al., 2007). A previous study suggested that oxidative stress, particularly in the hypothalamus, was involved in the activation of the SNS in obesity-induced hypertensive rats (Nagae et al., 2009). Recently, we also have demonstrated that AT_1_ receptor/NAD (P) H oxidase-induced oxidative stress in the RVLM causes sympathoexcitation in obesity-induced hypertensive rats (Kishi et al., 2011a; Konno et al., 2012). Leptin is reported to have an interaction with brain renin-angiotensin system in the regulation of the SNS (Hilzendeger et al., 2012).

These findings with regard to the salt-induced and/or obesity-induced hypertension strongly suggest that oxidative stress in the RVLM is increased not only by gene background (such as in SHRSPs or SHRs) but also by environmental factor. A recent study reported the epigenetic alteration is occurred in salt-induced hypertension (Mu et al., 2011). There is a possibility that various genetic, environmental, and epigenetic factors affect the brain and causes sympathoexcitation via oxidative stress in the RVLM.

## Effects of AT_1_ receptor blockers on oxidative stress in the brain and sympathetic nerve activity

As described above, the up-regulation of AT_1_ receptor in the brain has an important role in the pathophysiology of hypertension (Sved et al., 2003; Guyenet, 2006; Dupont and Brouwers, 2010) It is interesting to note that AT_1_ receptor is rich in the specific brain, such as, anteroventral third ventricle, PVN of the hypothalamus, NTS and RVLM in the brainstem (Allen et al., 1999; Hu et al., 2002; McKinley et al., 2003; Reja et al., 2006). It has been demonstrated that peripherally administered AT_1_ receptor blockers could penetrate the blood-brain barrier and blocks AT_1_ receptor within the brain as well as outside of the brain, although, the extent of the blocking action within the brain varies among AT_1_ receptor blockers when they are administered peripherally (Wang et al., 2003; Kishi et al., 2012b; Konno et al., 2012). The peripheral treatment with AT_1_ receptor blockers attenuates or nearly blocks the pressor response to centrally administered angiotensin II (Tsuchihashi et al., 1999; Nishimura et al., 2000; Gohlke et al., 2001; Kishi et al., 2012b; Konno et al., 2012) This is also observed with the microinjection of angiotensin II into the RVLM in SHRs orally treated with olmesartan (Lin et al., 2005). Furthermore, the central nervous system blockade by the peripheral administration of AT_1_ receptor blockers has been documented by autoradiographic binding studies (Wang et al., 2003). It should be noted that the high density of AT_1_ receptor is present in brain regions that are involved in the regulation of the SNS such as the circumventricular organs (for example, the SFO, the organum vasculosum laminae terminalis, and area postrema) outside of the blood-brain barrier where peripherally administered AT_1_ receptor blockers are able to access without considering the existence of the blood-brain barrier as well as inside of the blood-brain barrier (McKinley et al., 2003). Recent studies suggest that the systemic administered AT_1_ receptor blockers also act on the AT_1_ receptor within the brain, thereby reducing blood pressure in hypertensive rats (Tsuchihashi et al., 1999; Leenen and Yuan, 2001; Lin et al., 2005; Araki et al., 2009; Hirooka et al., 2010; Kishi et al., 2012b; Konno et al., 2012). The extent of the actions of AT_1_ receptor blockers within the brain might depends partly on the lipophilicity and pharmacokinetics (Gohlke et al., 2001; Wang et al., 2003). Sympathoinhibitory effect via reduction of oxidative stress through the inhibition of the AT_1_ receptor in the brain is differed between AT_1_ receptor blockers. Orally administered telmisartan or olmesartan reduced blood pressure and urinary norepinephrine excretion in SHRSPs, and it was associated with a reduction of oxidative stress production in the brainstem including the RVLM (Araki et al., 2009; Hirooka et al., 2010). Orally administered telmisartan could inhibit AT_1_ receptor-induced oxidative stress in the RVLM and activation of the SNS in SHRSPs to a greater extent than candesartan, in spite of similar depressor effects (Kishi et al., 2012b). In obesity-induced hypertensive rats, telmisartan could also inhibit AT_1_ receptor-induced oxidative stress in the RVLM and activation of the SNS to a greater extent than losartan, in spite of similar depressor effects (Konno et al., 2012). Thus, it is conceivable that orally administered AT_1_ receptor blockers might block the AT_1_ receptor in the brain, particularly in the RVLM, thereby reducing the oxidative stress production and reducing blood pressure via inhibiting the activation of the SNS.

## Sympathoinhibition by targeting brain oxidative stress in hypertension

3-hydroxy-3-methylglutaryl coenzyme A reductase inhibitors (statins) are potent inhibitors of cholesterol biosynthesis. Interestingly, a previous study suggests that statins reduce blood pressure in patients with hypertension (Golomb et al., 2008). Moreover, the potential sympathoinhibition of statins has been demonstrated (Kishi et al., 2003; Sinski et al., 2009; Gomes et al., 2010; Kishi and Hirooka, 2010; Siddiqi et al., 2011). It has been demonstrated that statins have anti-oxidant effect (Wassmann et al., 2002). We have demonstrated that orally administered atorvastatin causes sympathoinhibition and improves the impaired baroreflex sensitivity via reduction of oxidative stress through the inhibition of AT_1_ receptor-NAD (P) H oxidase and up-regulation of MnSOD in the RVLM of SHRSPs (Kishi et al., 2008, 2009, 2010b). Other previous studies have demonstrated that simvastatin normalized the activation of the SNS in rabbits with heart failure (Pliquett et al., 2003; Gao et al., 2005). However, it has not been fully clarified whether the statin-induced sympathoinhibition via reduction of oxidative stress in the brain is a class-effect or not.

Several calcium channel blockers have been confirmed to cause sympathoinhibition via reduction of oxidative stress in hypertensive rats. Orally administered amlodipine (Hirooka et al., 2006) or azelnidipine (Konno et al., 2008) cause sympathoinhibition via reduction of oxidative stress in the RVLM of SHRSPs. We confirmed that orally administered azelnidipine inhibit the NAD (P) H oxidase activity and activate MnSOD in the RVLM of SHRSPs (Konno et al., 2008). These results are consistent with previous study (Umemoto et al., 2004). Furthermore, combination of atorvastatin and amlodipine (Kishi and Sunagawa, 2012) or combination of olmesartan and azelnidipine (Shinohara et al., 2012) has additive effects of sympathoinhibition via reduction of oxidative stress in the brain.

Interestingly, calorie restriction (Kishi et al., 2011a) or exercise training (Kishi et al., 2012a) has a pivotal role to cause sympathoinhibition via reduction of oxidative stress through the inhibition of the AT_1_ receptor in hypertensive rats. These results provide us the possibility that adipocytokines and/or insulin resistance might affect the brain AT_1_ receptor, and cause sympathoexcitation.

## Perspectives

**Figure 1:**
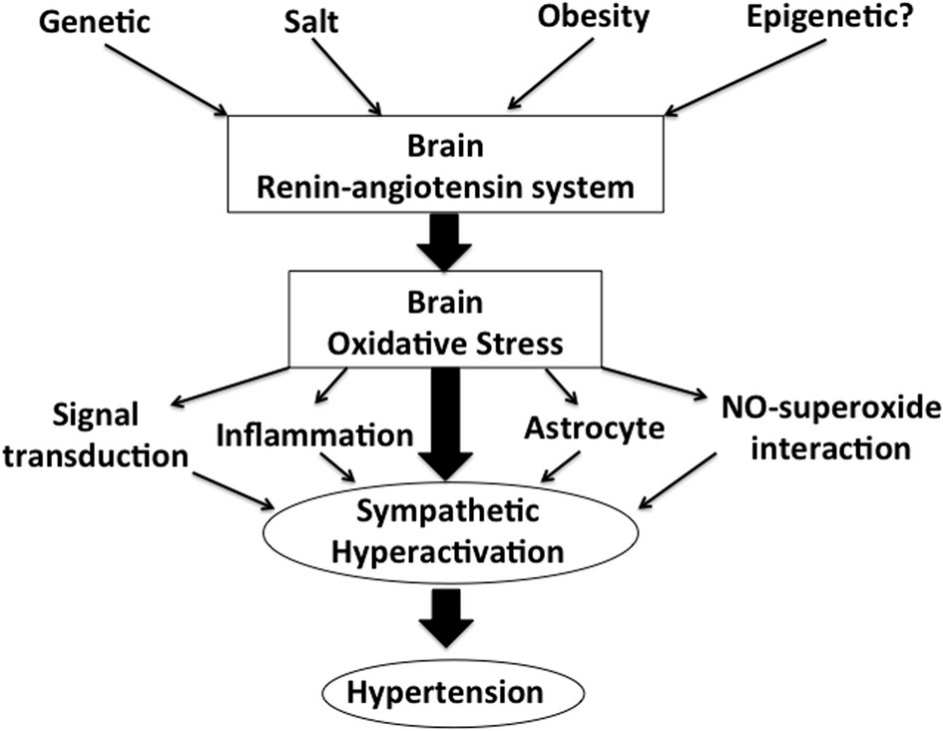
**A schema showing our concept in the regulation of sympathetic nerve activity via brain renin-angiotensin system and oxidative stress**.

Figure shows our concept in the regulation of the activation of the SNS via brain renin-angiotensin system and oxidative stress (Figure 1). In the brain, particularly in the autonomic regulatory regions, such as the RVLM, NTS and PVN of the hypothalamus, previous studies have suggested that the inhibition of the brain AT_1_ receptor may have a significant role in the sympathoinhibitory effect via reduction of oxidative stress. AT_1_ receptor blockers are widely used in the treatments for hypertension (Iwanami et al., 2009). It is also suggested that AT_1_ receptor blockers may have protective effects on neurons, reducing the incidence of stroke and improving cognition (Iwanami et al., 2009; Mogi and Horiuchi, 2009). In addition, renal afferent nerves may also contribute to the blood pressure elevation according to the recent findings of the renal nerve ablation in patients with resistant hypertension (Krum et al., 2009; DiBona and Esler, 2010; Simplicity HTN-2 Investigators, 2010). Renal afferent nerves project directly into many areas in the central nervous system controlling the activation of the SNS such as the NTS and hypothalamus (Calaresu and Ciriello, 1981; Ciriello and Calaresu, 1983; Stella et al., 1987). It is demonstrated that oxidative stress mediates the stimulation of the SNS in the phenol renal injury model of hypertension in which the renal afferent nerves are stimulated (Ye et al., 2006). In this model, brain AT_1_ receptor and NAD (P) H oxidase are activated. Previous studies have suggested that the increased oxidative stress production and reduced neuronal NOS expression may be involved in this mechanisms, which leads to the alteration of cytokines in the brain (Campese et al., 2005; Ye et al., 2006). It is interesting and important to consider that AT_1_ receptor and related oxidative stress production in the brain are as novel therapeutic targets of the treatments for hypertension.

### Conflict of interest statement

The authors declare that the research was conducted in the absence of any commercial or financial relationships that could be construed as a potential conflict of interest.
